# Distinguishing the Charge Trapping Centers in CaF_2_-Based 2D Material MOSFETs

**DOI:** 10.3390/nano14121038

**Published:** 2024-06-16

**Authors:** Zhe Zhao, Tao Xiong, Jian Gong, Yue-Yang Liu

**Affiliations:** 1 School of Physical Science and Technology, Inner Mongolia University, Hohhot 010021, China; zhezhao@mail.imu.edu.cn; 2 State Key Laboratory of Superlattices and Microstructures, Institute of Semiconductors, Chinese Academy of Sciences, Beijing 100083, China; xiongtao@semi.ac.cn; 3 Ordos Institute of Technology, Ordos 017000, China

**Keywords:** CaF_2_, 2D material MOSFETs, reliability, charge trapping

## Abstract

Crystalline calcium fluoride (CaF_2_) is drawing significant attention due to its great potential of being the gate dielectric of two-dimensional (2D) material MOSFETs. It is deemed to be superior to boron nitride and traditional silicon dioxide (SiO_2_) because of its larger dielectric constant, wider band gap, and lower defect density. Nevertheless, the CaF_2_-based MOSFETs fabricated in the experiment still present notable reliability issues, and the underlying reason remains unclear. Here, we studied the various intrinsic defects and adsorbates in CaF_2_/molybdenum disulfide (MoS_2_) and CaF_2_/molybdenum disilicon tetranitride (MoSi_2_N_4_) interface systems to reveal the most active charge-trapping centers in CaF_2_-based 2D material MOSFETs. An elaborate Table comparing the importance of different defects in both n-type and p-type devices is provided. Most impressively, the oxygen molecules (O_2_) adsorbed at the interface or surface, which are inevitable in experiments, are as active as the intrinsic defects in channel materials, and they can even change the MoSi_2_N_4_ to p-type spontaneously. These results mean that it is necessary to develop a high-vacuum packaging process, as well as prepare high-quality 2D materials for better device performance.

## 1. Introduction

Two-dimensional (2D) materials offer new possibilities for advancing Moore’s Law due to their ultra-thin thickness and smooth surface with no dangling bonds [1,2,3,4,5,6,7,8,9]. The ultra-scaled channel places higher demands on the quality and reliability of gate dielectric materials. However, common oxides (such as SiO_2_ [10], hafnium dioxide (HfO_2_) [11], and aluminum trioxide (Al_2_O_3_) [12]) that are used in silicon technologies are non-layered, which makes it difficult for them to form a good interface with 2D channels. To deal with the problem, 2D dielectrics such as hexagonal boron nitride (h-BN) have been studied [13]. However, the band gap (~6 eV) and dielectric constant (5.06 ε) of h-BN are not satisfying for dielectric materials [14]. Its band offset with 2D materials is not large enough, which will lead to many reliability problems [15].

Recent experimental preparation of crystalline CaF_2_ provides a promising solution to the dilemma [16,17]. By using molecular beam epitaxy (MBE), crystalline CaF_2_ can be grown on a silicon or germanium substrate [18]. It has a larger bandgap (12.1 eV) and dielectric constant (8.43 ε) than h-BN [19]. The grown CaF_2_ is terminated by F atoms, which means that there are no dangling bond on its surface [20]. At the same time, wafer-scale CaF_2_ was prepared by the magnetron sputtering method as a substrate for optoelectronic devices, resulting in the formation of good van der Waals devices with Tin disulfide (SnS_2_) and Tungsten disulfide (WS_2_). The electronic mobility and photoresponsivity of the devices were improved by an order of magnitude higher compared to SiO_2_-based devices [21]. Another important point is that CaF_2_ itself is stable in air, and is not easily dissolved in water [22]. CaF_2_ can form good I-band alignment with many 2D materials, such as silicon carbide (SiC). The valence band offset of 2D SiC/CaF_2_ is as high as 3.5 eV, and even if there are carbon antisite and interstitial defects on the 2D SiC surface, it will not affect CaF_2_ [23]. This means that it will be very advantageous as a gate dielectric for semiconductor devices.

Nevertheless, notable device reliability issues were still observed in CaF_2_-based MOSFETs [19,22,24,25], which contradicts the perfect electrical properties of CaF_2_. For example, the *I*_D_-*V*_G_ hysteresis is significant (although, lower than that in MoS_2_/SiO_2_ FET), and it shows obvious variability when the same device is operated at different scanning times. On the other hand, when different devices are operated under the same *V*_D_, the *I*_D_-*V*_G_ characteristics such as on/off current ratio and subthreshold swing (SS) (150–90 mV dec^−1^) differ greatly [19]. In addition, some devices with large negative threshold voltage (*V*_th_) are prone to fail due to the bias overload of the CaF_2_ layer. The physical origin of hysteresis and threshold voltage shift is widely attributed to the charge trapping and de-trapping of microscopic defects [26,27,28,29,30,31,32], and the strength of the charge trapping effect is closely related to the type of defects [33,34,35,36]. In graphene/CaF_2_ FETs, the hysteresis and bias–temperature instabilities (BTI) phenomenon are both observed due to the presence of defects. They are not detrimental to device performance due to the intrinsic advantage of CaF_2_, but the problem cannot be avoided [37]. The hysteresis is also observed in ReS_2_ FETs, and it is subjected to variations in temperature, sweeping gate voltage, and pressure during experiments, demonstrating the existence of a charge-trapping and de-trapping effect [38].

The presence of trapping centers at the interface not only affects the reliability of transistors, but also has an impact on other kinds of semiconductor devices, such as thermoelectric devices composed of tin dioxide (SnO_2_) [39] and solar cell devices composed of perovskite materials such as perovskite solar cells (PSCs) [40]. Therefore, distinguishing active trapping centers, and then finding ways to eliminate them, is crucial for the improvement of semiconductor devices. Unfortunately, it is difficult to determine the specific contribution of each kind of defect to the charge-trapping process through experiments. Under such circumstances, we decides to use principles calculations to distinguish the active charge-trapping centers in CaF_2_-based 2D MOSFETs first, and then provide guidance to experimental researchers to analyze and improve the performance and reliability of their devices.

In this work, realistic MoS_2_/CaF_2_ and MoSi_2_N_4_/CaF_2_ interface models have been constructed to study the charge-trapping centers in various positions. CaF_2_ is designed as a 5-layer structure, which is consistent with the experimental report [19,41]. The fabricated device in the experiment contains a 2-layer MoS_2_ and a 2 nm thick CaF_2_, which is 5 layers. At the same time, 13 types of defects were systematically investigated, and several positions for each type of defect were studied to avoid randomness. When analyzing defects, we not only considered the defect energy levels, but also the defect formation energy and their importance in n-type and p-type transistors, respectively. To ensure the accuracy of the data, Heyd–Scuseria–Ernzerhof (HSE) hybrid functionals were used, even though they require a large amount of computing resources.

## 2. Materials and Methods

Among the 2D materials, MoS_2_ is one of the most widely used semiconductors [42,43,44,45]. It has a direct band gap of 1.8 eV, and has been used to design high-performance electronic and optoelectronic devices [5]. On the other hand, there are also some new materials being synthetized, such as the MoSi_2_N_4_ [46]. MoSi_2_N_4_ is very promising because of the excellent photocatalytic performance [47], mechanical strength [48], and electrical transportability [49]. Therefore, we construct both MoS_2_/CaF_2_ and MoSi_2_N_4_/CaF_2_ interface models to make the simulation results representative. The lattice parameter of CaF_2_, MoS_2,_ and MoSi_2_N_4_ is 3.90 Å, 3.16 Å, and 2.91 Å, respectively. To achieve good lattice matching, the primary cell of MoS_2_ is repeated five times to contact the CaF_2_ cell, which is repeated four times. The final CaF_2_ deformation is only 1.28%. Similarly, the primary cell of MoSi_2_N_4_ is repeated four times to contact the CaF_2_, while the CaF_2_ deformation is repeated three times and is only 0.52%.

To make the results reliable, different types of defects/impurities, not only within the material, but also at the interfaces and surfaces, were studied. For CaF_2_, even though previous studies have shown that it only contains a very small number of F defects (V_F_), for the sake of data reliability, research was still conducted on V_F_ defects. Meanwhile, our research found that V_F_ contributes two electrons to CBM, which had not been discovered by previous researchers. For MoS_2_, we considered S vacancy defect (V_S_), Mo vacancy defects (V_Mo_), MoS_3_ vacancy defect (V_MoS3_) and MoS_6_ vacancy defect (V_MoS6_) at different spatial locations. MoS_2_ is composed of one Mo atom in the middle and three S atoms on the upper and lower surfaces. A MoS_3_ defect is defined as the loss of a Mo atom and three S atoms connected to it, either in the upper or lower layers. The MoS_6_ defect is formed by the loss of both the Mo atom in the middle and the six S atoms connected to it. On the other hand, considering that gas adsorption is occurs very easily in the process of device manufacturing, we also studied the water and oxygen molecules that adsorbed at different positions. For a more intuitive display of defects and adsorption, the related structural diagrams are shown in following figures. For MoSi_2_N_4_, both its N vacancies (V_N_) and Si vacancies (V_N_) were studied simultaneously. Same as MoS_2_, gas adsorption in MoSi_2_N_4_ during preparation is also a factor that may affect device stability. The adsorption of O_2_ and water molecules (H_2_O) was studied in CaF_2_-MoSi_2_N_4_.

**Figure 1:**
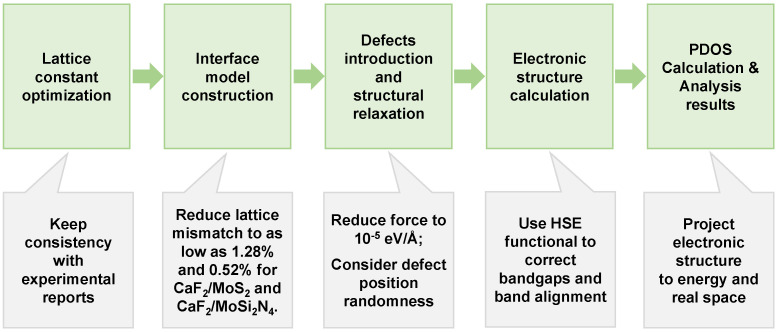
Flowchart of calculation method.

All the first-principles calculations were performed by the software PWmat [50,51]. The SG15 pseudopotential [52] was adopted, and the plane wave cutoff energy was 50 Ry. The Perdew–Burke–Ernzerhof (PBE) functional was used for structural relaxation with a convergence criterion of 10^−5^ eV/Å. The HSE [53] functional was used in the calculation of electronic structures to improve the accuracy of calculations. All calculations were performed using gamma points (0,0,0) considering the largeness of the supercells, and this is a common strategy to deal with large models [34,35]. VdW-D3 was used to correct the interlayer interaction of the material. The DFT-D3 energy formula is as follows: $E_{DFT-D3}=E_{KS-DFT}-E_{disp}$, $E_{KS-DFT}$ is the usual self-consistent KS energy and $E_{disp}$ is the dispersion correction as a sum of two- and three-body energies [54]. The equilibrium distance between the MoS_2_ and CaF_2_ and between the CaF_2_ and MoSi_2_N_4_ was 2.89 Å and 2.93 Å, respectively. For MoS_2_, the impact of point defects on the equilibrium distance was not significant, only 1.04%. For larger defects, there may have been some impacts, among which V_MoS3_ decreased the distance by 8.65% to 2.64 Å. O_2_ adsorption resulted in an equilibrium distance of 3.03 Å, which represented an increase of 5.21%. For MoSi_2_N_4_, the V_N_ defect showed a change in the equilibrium distance between CaF_2_-MoSi_2_N_4_, with an equilibrium position of 2.72 Å, representing a 7.17% decrease. H_2_O adsorption resulted in an equilibrium position of 3.10 Å, which represented an increase of 5.80%. The data above show that defects and adsorption can slightly change the equilibrium distance between interfaces, but their impact is not significant. All the calculation processes are shown in Figure 1.

## 3. Results

### 3.1. The Charge-Trapping Centers in CaF_2_-MoS_2_

**Figure 2:**
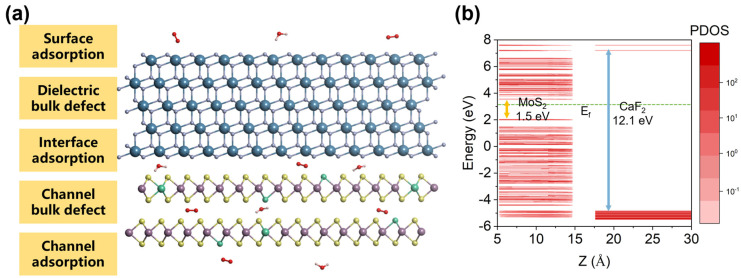
Atomic structure and type-I band alignment of CaF_2_-MoS_2_ interface models. (**a**) Atomic structure of 5-layer CaF_2_ and 2-layer MoS_2_, as well as (**b**) band alignment along the *Z*-axis direction.

The CaF_2_-MoS_2_ interface models are shown in Figure 2a. Blue, gray, purple, yellow, white, and red spheres are used in the figure to represent Ca, F, Mo, S, H, and O atoms. Figure 2a shows the adsorption and defects (green spheres) present at different interfaces and surfaces of CaF_2_-MoS_2_. A 5-layer CaF_2_ is adopted because the experimental MBE grown CaF_2_ is about 2 nm thick. The band alignments that manifested by the projected density of states (PDOS) are shown in Figure 2b. The red part in the figure represents the data of DOS, and the depth of the color represents the size of PDOS values. It can be seen that the VBM (valence band maximum) and CBM (conduction band minimum) are provided by MoS_2_, and the band offsets are greater than 2 eV, which makes charge tunneling difficult. All Fermi energy levels have been reset to zero, indicated by a green dotted line in the graph. The defect energy level and band offset have a direct impact on the charge-trapping activity. Although the vacuum levels were not adjusted, this does not affect the conclusions reached. This confirms that using CaF_2_ as the gate of 2D material MOSFETs is likely to obtain good device reliability [41]. Therefore, when considering practical applications, we believe that the reliability issues should stem from some intrinsic or external charge-trapping centers.

#### 3.1.1. The Charge-Trapping Centers in CaF_2_

**Figure 3:**
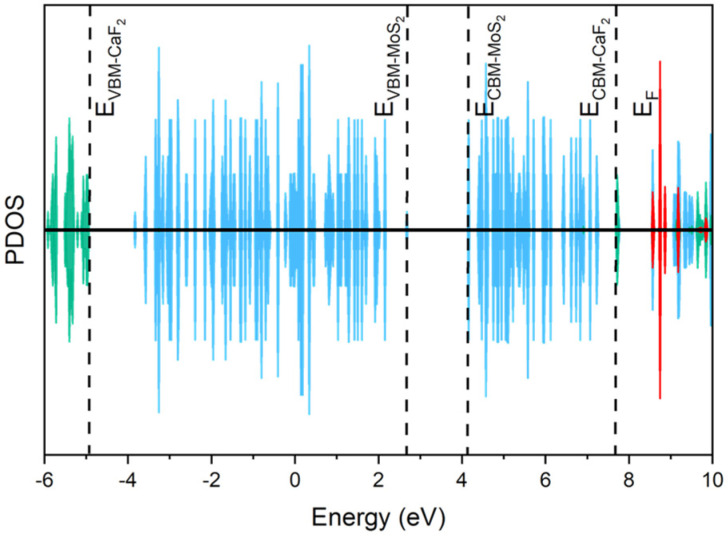
The position of the F vacancy (V_F_) defect energy level in the CaF_2_ band. The blue, green, and red lines represent the PDOS of CaF_2_, MoS_2_, and V_F_, respectively.

Intuitively, we should first study the F vacancy defect in the CaF_2_ layer. However, it has been demonstrated in experiments that generating defects in CaF_2_ is not easy [19]. Furthermore, it has been proven by a first-principle calculation that even though F vacancies (V_F_) and Ca vacancies (V_Ga_) exist, there is no defect state near the band edge of channel material due to the large band offset between the two materials [55]. Nevertheless, to make the conclusion more rigorous, we still conducted relevant calculations on the V_F_. In Figure 3, the energy levels of CaF_2_, MoS_2_, and V_F_ are represented by green, blue, and red, respectively. In the calculation, both vdW and electron spin are considered, and the randomness of V_F_ positions is also taken into account. For ease of observation, the PDOS value of V_F_ in Figure 3 has been expanded 50 times. As the focus is on the defect energy level of V_F_, it does not affect the results. The band alignment of CaF_2_ and MoS_2_ here is consistent with Figure 2b, and MoS_2_ provides VBM and CBM. The offset between the V_F_ defect energy level and CBM is 4.43 eV, indicating that even with defects, it is not easy to trap charges. Consequently, we turn our attention to the trapping centers inside the channel material, in the semiconductor/dielectric interface, and at the dielectric surface.

#### 3.1.2. The Charge-Trapping Centers in the Channel

**Figure 4:**
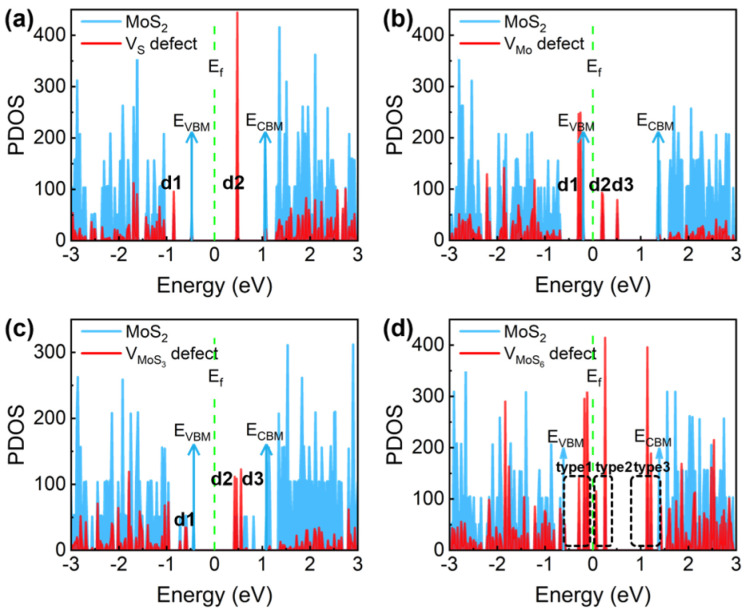
The energy level distribution of different defects. (**a**) S vacancy (V_S_), (**b**) Mo vacancy (V_Mo_), (**c**) MoS_3_ vacancy (V_MoS3_), and (**d**) MoS_6_ vacancy (V_MoS6_).

The energy level distribution of different defects in MoS_2_ is shown in Figure 4. First, in Figure 4a, there is an occupied defect state denoted by d1 for the vs. in MoS_2_, whose energy is 0.38 eV below VBM, and there are two empty defect states with similar energy denoted by d2, whose energy is 0.57 eV below CBM. According to charge transfer theories, the charge-trapping rate will decrease exponentially with the increasing energy barrier between the initial and final electronic states; thus, we can consider that only the defect levels located less than 1 eV away from the MoS_2_ band edge are active trapping centers. Therefore, it can be concluded that d1 is an important hole-trapping state in p-FETs, and d2 is an important electron-trapping state in n-FETs. Similarly, in Figure 4b, the Mo vacancy is active in trapping holes and electrons, but not as active as the S vacancy in electron trapping because the V_Mo_ defect levels are farther away from the CBM. In addition to the common vs. and V_Mo_, experiments have reported that complex vacancy defects (such as V_MoS3_ and V_MoS6_, as shown in Figure 4c and Figure 4d, respectively) are found in MoS_2_ [56]. These two complex vacancies contain many dangling bonds, and thus, can introduce a series of defect states (up to 13) located either close to VBM or to CBM. Consequently, they will be very active charge-trapping centers. However, the energy of the formation of these complex defects is very high, resulting in a low density. More details of the defect levels have been listed in Table 1.

#### 3.1.3. The Charge-Trapping Centers in the Interface and Surface

**Figure 5:**
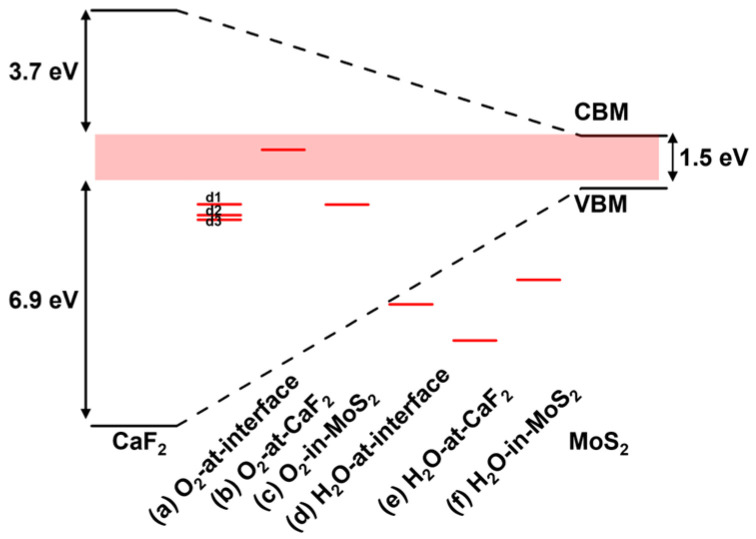
The energy level distribution of different molecules adsorbed on the surface and interface of CaF_2_-MoS_2_.

It has been mentioned in previous reports that the hysteresis of CaF_2_-MoS_2_ devices can be reduced after they are heated and dried [19]. This indicates that molecules had been adsorbed during device preparation, so the activity of these adsorbates needs to be discussed. Figure 5 shows the adsorption of O_2_ at the CaF_2_-MoS_2_ interface, and three defect levels denoted by d1, d2 and d3 are observed. They are only 1 eV, 0.85 eV and 0.54 eV below VBM, respectively. Therefore, they will be active hole traps in p-MOSFETs. In contrast, the adsorption of water molecules at the interface is much less important because they do not induce obvious defect states near the band edge of MoS_2_.

**Figure 6:**
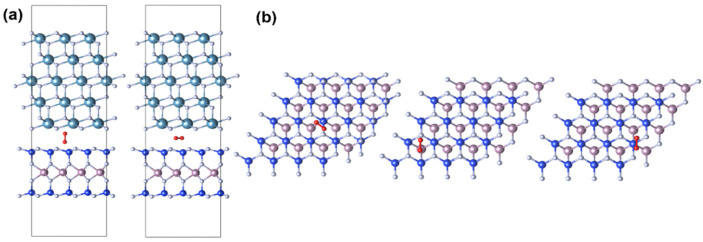
Different situations of O_2_ adsorption. (**a**) Compare O_2_ perpendicular/parallel to the interface; (**b**) compare different adsorption positions.

In discussing the adsorption of O_2_, we first tested different placement methods, including those parallel and perpendicular to the interface, as shown in Figure 6a. To ensure the reliability of our conclusion, we tested O_2_ at three different positions, as shown in Figure 6b. The CaF_2_ layer was removed from the atomic schematic for ease of observation. Moreover, all of our defects and adsorption structures were tested in at least three different locations to prevent randomness. All results demonstrate the reliability of the existing data. To further check the importance of oxygen, we studied the oxygen that adsorbed in other positions. Figure 5 shows the situation where oxygen molecules are adsorbed in the interlayer of MoS_2_. It can be seen that the defect state is only 0.37 eV below VBM, which will trap holes easily, and thus, affects the device performance. Figure 5 shows the case where oxygen is adsorbed on the surface of CaF_2_. An occupied defect state that is close to CBM rather than CBM is seen. Considering that the negative gate voltage in a p-FET will drag the defect level down toward the VBM, the oxygen on the CaF_2_ surface will form very active hole-trapping centers with large gate voltage.

To exhibit the importance of different defects more clearly, Table 1 summarizes the information of all defects. The defect levels that are more than 1 eV away from the MoS_2_ band edge are regarded as electronically unimportant [57,58,59]. The Δ*E_VBM/CBM_* is calculated as; moreover, the formation of energy/adsorption energy is considered to provide an overall evaluation of their importance.

### 3.2. The Charge-Trapping Centers in CaF_2_-MoSi_2_N_4_

**Figure 7:**
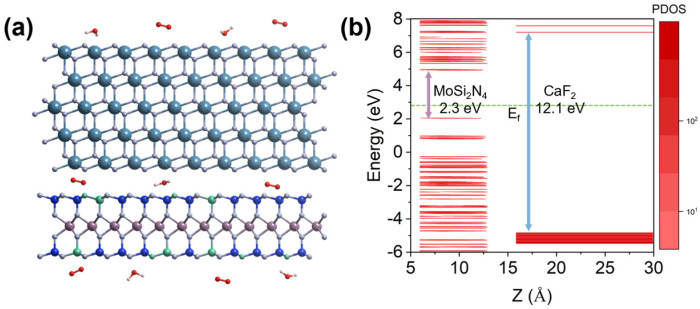
Atomic structure and type-I band alignment of CaF_2_-MoS_2_ interface models. (**a**,**b**) The atomic structure of 5-layer CaF_2_, and 1-layer MoSi_2_N_4_, as well as the band alignment along the *Z*-axis direction.

Now, we study the MoSi_2_N_4_-CaF_2_ system. MoSi_2_N_4_ is a 2D material with seven atomic layers. One Mo atomic layer lies in the middle while two Si-N-Si tri-layers lie on the top and bottom surfaces symmetrically. It can be seen that the VBM and CBM are provided by MoSi_2_N_4_ (Figure 7b), and the band offsets are greater than 2 eV, which makes charge tunneling difficult. Vacancy defects caused by the shedding of N atoms and Si (Figure 7a) atoms on the surface layer are the primary problems to be considered. At the same time, the influence of the adsorption of oxygen molecules and water molecules (Figure 7a) during device manufacture is also considered. The atoms highlighted in green in Figure 7a represent defects and adsorption sites.

**Figure 8:**
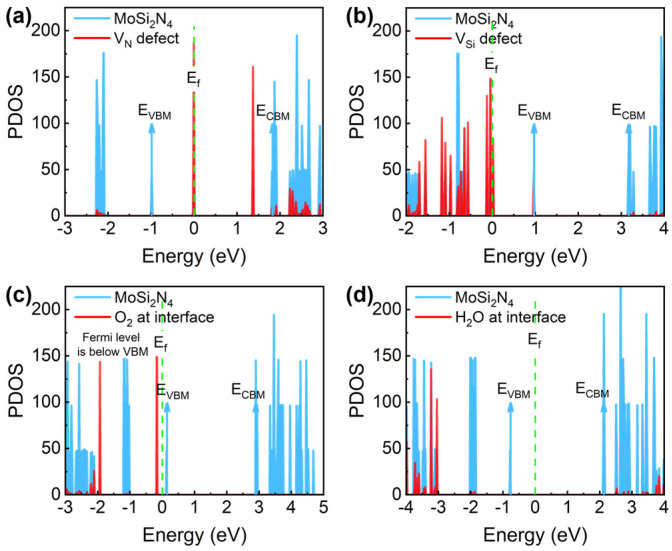
The energy level distribution of different molecules adsorbed on the surface and interface of CaF_2_-MoS_2_. (**a**) N vacancy (V_N_), (**b**) Si vacancy (V_Si_), (**c**) O_2_ at interface, and (**d**) H_2_O at interface.

For the N vacancy (V_N_) (Figure 8a), two defect levels are induced into the band gap, of which the half-occupied d1 state is 0.98 eV above VBM and the empty d2 state is 0.45 eV below CBM. Such small energy barriers make them very active hole/electron-trapping centers. In contrast, the V_Si_ defect induces no defect levels close to the CBM, as is shown in Figure 8b, but it induces many defect levels below the VBM. Specifically, the electrons in VBM have spontaneously transferred to the defect sites, shifting the Fermi level below the VBM and making the CaF_2_-MoSi_2_N_4_ a whole p-type heterostructure. Interestingly, the adsorption of oxygen in the CaF_2_-MoSi_2_N_4_ interface has a very similar effect, as is shown in Figure 8c, the electrons in VBM are spontaneously captured by the oxygen, and the MoSi_2_N_4_ becomes a p-type material. If the oxygen density is high, the performance and reliability of the device will be greatly reduced. In comparison, the adsorption of water molecules in the interface does not have such an effect, as is shown in Figure 8d. The water-related defect energy level is far from the band edge of MoSi_2_N_4_. This further confirms that water molecule adsorption is less important than oxygen adsorption in impacting device performance and reliability. To present the importance of different defects more intuitively, Table 2 summarizes and compares the information of all defects in the CaF_2_-MoSi_2_N_4_ system.

## 4. Conclusions

In conclusion, we have investigated the various defects and adsorbates in CaF_2_-based 2D material MOSFET structures to distinguish their importance in degrading device performance and reliability. First, the intrinsic defects in channel materials, including the Vs. and V_Mo_ in MoS_2_, and V_Si_ and V_N_ in MoSi_2_N_4,_ are very active charge-trapping centers. At the same time, although the intrinsic defect V_MoS6_ causes many defect states in the band gap, it is not a significant defect due to its large formation energy. Second, the adsorbed oxygen molecules in the channel/CaF_2_ interface or CaF_2_ surface are very important trap centers, and they can even spontaneously change the MoSi_2_N_4_ to p-type. Third, the adsorbed water molecules are inactive in capture charges, and thus, are much less important in affecting device performance. An elaborate table comparing the detailed properties of different defects is provided so that both experimental researchers and theorists can refer to it easily. Moreover, the intrinsic defect V_Si_ in CaF_2_-MoSi_2_N_4_ can also lead to conversion to p-type transistors. Finally, we found that V_F_ in CaF_2_ spontaneously contributes two electrons to CBM.

The significance of defects or adsorption in CaF_2_-based 2D material MOSFETs is not solely contingent upon the defect energy level; rather, it is also contingent upon the formation energy and transport type of the device. The two tables presented in the article provide a comprehensive demonstration of the impact of defects on performance. Furthermore, this methodology can facilitate the development of a system tool in the future, which will enable the determination of the impact of defects on device performance. Especially worth mentioning is the adsorption of oxygen molecules, which is a more problematic phenomenon than the adsorption of water molecules. To avoid this issue, it is advisable to isolate oxygen as much as possible during device preparation or use objects that do not introduce additional pollution sources to adsorb oxygen. These results mean that the exclusion of adsorbates in device fabrication is as important as growing high-quality channel material to obtain better device performance. The findings of our research can be extrapolated to the significance of different capture centers in a variety of 2D material MOSFETs.

## Figures and Tables

**Table 1:** Importance of different trapping centers in CaF_2_-MoS_2_.

Defect Types	Defect State	ΔE- VBM (eV)	ΔE- CBM (eV)	n-FET Importance	p-FET Importance	Fromation Energy (eV)	Overall Importance
V_S_	d1	−0.38	−1.91	✓	✗	2.91	✓
	d2	0.95	−0.57	✓	✓		
V_Mo_	d1	−0.06	−1.63	✓	✗	8.52	✓
	d2	0.40	−1.17	✗	✓		
	d3	0.71	−0.86	✗	✓		
V_MoS3_	d1	−0.25	−1.78	✓	✗	11.81	✓
	d2	0.89	−0.64	✗	✓		
	d3	0.99	−0.53	✗	✓		
V_MoS6_	type1	<0.50	>1.50	✓	✗	21.41	✗
	type2	<1.00	>1.00	✗	✗		
	type3	>1.75	<0.25	✗	✓		
O_2_ at interface	d1	−0.99	−2.45	✓	✗	0.68	✓
	d2	−0.55	−2.00	✓	✗		
	d3	−0.85	−2.31	✓	✗		
H_2_O at interface		−3.42	−4.91	✗	✗	0.61	✗
O_2_ in MoS_2_		−0.37	−2.01	✓	✗	2.35	✓
O_2_ at surface		1.11	−0.41	✗	✓	2.25	✓

**Table 2:** Importance of different capture centers in CaF_2_-MoSi_2_N_4_.

Defect Types	Defect State	ΔE- VBM (eV)	ΔE- CBM (eV)	n-FET Importance	p-FET Importance	Fromation Energy (eV)	Overall Importance
V_N_	d1	0.98	−1.83	✓	✗	5.97	✓
	d2	2.36	−0.45	✗	✓		
V_Si_	d1	−1.01	−3.23	✗	✗	11.15	✓
	d2	0.00	−2.22	✓	✗		
O_2_ at interface		−0.32	−3.07	✓	✗	0.19	✓
H_2_O at interface		−2.29	−5.17	✗	✗	0.34	✗

## Data Availability

The data presented in this study are available on request from the corresponding author.
